# Ocular hypertension after EyeCee One preload lens implantation: a retrospective cohort study

**DOI:** 10.3389/fmed.2024.1402606

**Published:** 2024-07-23

**Authors:** Julio González-Martín-Moro, Yolanda Fernández Miguel, María Castro-Rebollo, Carlos Izquierdo-Rodríguez, Francisco Luis Prieto-Garrido, Victoria Padeira Iranzo, Vanesa Mittendrein, Vicente Miralles Pechuan, Alicia Ruiz-Pomeda, Rosario Cobo-Soriano

**Affiliations:** ^1^Department of Ophthalmology; Hospital Universitario del Henares, Fundación para la Investigación e Innovación Biomédica del Hospital Universitario Infanta Sofía y del Hospital Universitario del Henares (FIIB HUIS HHEN), Madrid, Spain; ^2^Department of Health Sciences; Universidad Francisco de Vitoria, Madrid, Spain; ^3^Department of Optometry and Vision, Faculty of Optics and Optometry, Universidad Complutense de Madrid, Madrid, Spain

**Keywords:** cataract surgery, EyeCee One, intraocular lens, glaucoma, intraocular pressure

## Abstract

**Objective:**

In 2022, several cases of ocular hypertension (OHT) related to EyeCee One preloaded IOLs were reported. The aim of this study was to determine the presurgical and surgical variables associated with this response.

**Methods and analysis:**

An analysis was conducted on patients who underwent isolated cataract surgery between September 2022 and December 2022 at the Hospital Universitario del Henares. The influence of potential factors was studied using the Kruskal–Wallis test and multiple regression analysis.

**Results:**

A total of 353 cataract surgeries were included in the study. No significant differences between the different IOLs were found related to a change in the IOP on the first postoperative day (*p* = 0.395), but the change in the IOP after 1 month was higher in the EyeCee One group (*p* = 0.016). Approximately 6.1% of the patients who received EyeCee One had an IOP increase greater than 10 mmHg, compared to only 0.8% of the patients who received other IOLs. The odds ratio (OR) of experiencing an IOP increase greater than 10 mmHg in the EyeCee One group at the 1-month visit was 7.99 (1.52–41.99). The multiple regression analysis showed that receiving the EyeCee One lens was associated with a 2-mmHg increase in IOP. A previous history of glaucoma or OHT was not associated with greater IOP. Two patients in the EyeCee One group developed severe visual loss.

**Conclusion:**

Patients who received the EyeCee One IOL experienced significant increases in IOP at the 1-month visit. A small number of patients might suffer visual loss secondary to the rise in IOP.

## 1 Introduction

During the last months of 2022, a number of cases of ocular hypertension (OHT) following uncomplicated cataract surgery were reported in Spain and other countries (1). These cases of unexplained postoperative OHT were later associated with the EyeCee One preloaded and the EyeCee One Crystal preloaded IOLs, manufactured by NIDEK and distributed by Bausch&Lomb. An internal investigation at NIDEK identified the coating agent used in the nozzle portion of the injector, polyvinylpyrrolidone (PVP), as the reason why the drainage pathway of aqueous humor was obstructed, leading to an increased intraocular pressure (IOP). These cases prompted the laboratory to halt distribution and issue a recall of these lenses (1). The EyeCee One IOLs used in our study were confirmed to be part of the affected batch identified by NIDEK (NIDEK, July 2023).

Various risk factors for acute OHT on the first day after cataract surgery have been suggested, such as incomplete removal of viscoelastic material, surgery performed by a resident, male patients, a prior history of glaucoma or pseudoexfoliation, axial length greater than 25 mm, poor pupillary dilation, tamsulosin use, or corticosteroid response (2, 3). However, to date, only one study has linked one model of intraocular lens (IOL) to changes in postoperative IOP values (1, 4).

Very few articles have been published on this topic, as these cases constitute a recently discovered condition (1). OHT is often asymptomatic and not always accompanied by pain, ocular inflammation, or significant corneal edema. While elevated IOP can sometimes lead to symptoms such as pain or visual disturbances, it may be detected during routine eye exams without any obvious clinical signs.

In the ophthalmology department of the Hospital Universitario del Henares, six different models of IOLs were implanted in 2022. We analyzed the postoperative IOP of the entire cohort of patients undergoing cataract surgery at our center during the 3 months prior to the withdrawal of the lens. The objective of this study is to determine whether the observed increase in IOP is an idiosyncratic response or if the entire cohort of patients receiving the EyeCee One lens experienced higher IOP after surgery. Additionally, the study aims to identify preoperative characteristics of patients and intraoperative variables that could be related to this atypical outcome.

## 2 Materials and methods

This study was conducted with the entire cohort of patients undergoing cataract surgery at the Ophthalmology Department of the Hospital Universitario del Henares from 1 September 2022 to 12 December 2022. This timeframe coincided with the period of higher incidence of this side effect in Spain, just before Bausch&Lomb and NIDEK decided to discontinue the commercialization of the IOL and to withdraw supplies from hospitals. The sample was created using convenience sampling, focusing on November, when the first cases were detected in our center. The Hospital Universitario del Henares is a level 1 hospital serving a population of approximately 200,000 inhabitants. Eleven experienced surgeons and two residents performed cataract surgery during the study period. Only isolated cataract surgery was considered in the study. Combined cataract surgery with vitrectomy or glaucoma was considered an exclusion criterion. Rupture of the posterior capsule with sulcus IOL implantation was also an exclusion criterion, as it involves the implantation of a different IOL model. Other complications, including the rupture of the posterior capsule, were not considered exclusion criteria, provided one of the usual in-the-bag-IOLs was implanted.

In our center, patients undergoing uncomplicated cataract surgery are typically seen the day after surgery and between 4 and 6 weeks later (1-month post-cataract surgery visit). If deemed necessary, the surgeon schedules intermediate visits. The first visit includes a slit lamp examination and IOP measurement using an air tonometer. The patient follows a treatment regime combining topical ofloxacin every 4 h during the daytime for the first week and topical dexamethasone, 1 drop every 4 h for the first week, gradually decreasing over 5 weeks. Additional medications, such as bromfenac, anti-edema ointment, or ocular hypotensives, may be added at the surgeon’s discretion. The 1-month visit, prior to discharge, includes IOP measurement, patient visual acuity, automated refraction, and a fundus examination. Goldman tonometry is only performed in cases where IOP is high, or the patient has a history of glaucoma.

To conduct the study, information from the digital surgical formularies was transferred into an Excel database. These surgical questionnaires capture all intraoperative information minutes after the surgical procedure has been completed, including epidemiological variables (age and gender), clinical variables (alpha-adrenergic blockers intake, drug allergies, and comorbidities), and variables related to the surgical procedure (type of anesthesia, surgical complications, use of trypan blue, intracameral use of phenylephrine or acetylcholine, type of intracameral antibiotic, IOL power, axial length, and anterior chamber depth). To complete the database, the electronic clinical charts of the patients were reviewed to add information regarding the presence of glaucoma, the number of antiglaucoma drugs, presurgical visual acuity, and postsurgical visual acuity in the previous month. Since most patients are not refracted before or after cataract surgery (if the visual result has been optimal), pinhole visual acuity was used as an approximate measure of the best-corrected distance visual acuity. IOP at the pre-cataract surgery appointment was considered baseline IOP. If this information was not available on the last visit before surgery, it was obtained from the nearest visit that included it. IOP was also measured on the first day and at the 1-month post-cataract surgery visit. Since IOP after cataract surgery in our center is usually measured using air tonometry, air tonometry data were preferred if both air tonometry and applanation tonometry records were available. Nuclear cataract grade (assessed using the LOCS III classification) was also recorded and considered a surrogate variable of cataract severity.

During the study period, in addition to the EyeCee One, other five models of IOL were implanted: PhysIOL® 123 Micropure IOL, J&J® Tecnis Eyhance DIB00, the Alcon® ACU00T0, Alcon® SN6CWS, and Alcon® AcrySof Toric SN6ATx.

The information, along with data obtained from surgical protocols, was entered into an Excel database and analyzed using SPSS (SPSS 22, IBM Corporation). The main variables were the change in IOP on the first day after surgery (first day IOP - baseline IOP) and the change of IOP at the 1-month visit (1-month visit IOP - baseline IOP). The normality of the variables was assessed using the Kolmogorov–Smirnov test. The three IOLs marketed by Alcon (ACU00T0, SN6CWS Alcon®, and AcrySof Toric SN6ATx Alcon®), which share the same platform and material, were grouped for statistical analysis and optimization. There were two main dependent variables: the change in IOP compared to preoperative IOP on the first postoperative day and at the 1-month visit. The Kolmogorov–Smirnov test showed that neither of the two variables followed a normal distribution, so a non-parametric approach was carried out.

Demographic variables were expressed in terms of means and standard deviations, except for visual acuity, which, as measured using a decimal scale, is non-parametric and therefore was expressed as median and interquartile range. Changes in IOP were expressed both ways. The Kruskal–Wallis test was used to initially compare the four IOL models. In a second approach to increase statistical power, the change in pressure experienced by the eyes that received the EyeCee One implant was compared to a group containing eyes that received any of the other IOLs.

The influence of potential confounding variables in the association between the studied IOL model and IOP was studied using multiple regression analysis. The possible association of the change in IOP with the IOL model was also graphically analyzed using box plots. The number of patients that experienced IOP elevations exceeding 5 and 10 mmHg from baseline was determined, and tables were created to assess the risk of peak IOP exceeding 5 and 10 mmHg associated with the EyeCee One implant.

The study was conducted in accordance with the Declaration of Helsinki after obtaining approval from the clinical research committee of the University Hospital of La Princesa. (Study Number: 5237).

## 3 Results

During the 15 weeks of the study, 355 cataract surgeries were performed on 349 patients. Only six patients underwent cataract surgery on both eyes during the period of study. In these six cases, cataract surgery was performed on different days. Two eyes were excluded from the study due to posterior capsule rupture that required the implantation of a three-piece IOL in the sulcus and thus the final number of patients included in the analysis was 353. Table 1 shows the presurgical characteristics and intraoperative variables of the eyes included in the study. The average age of the patients was lower in the group that received the J&J® Tecnis Eyhance DIB00 and PhysIOL® Micropure 123 IOLs. Preoperative visual acuity, nuclear cataract grade, and biometric values were similar in all four groups. All patients, except 8, underwent surgery with topical anesthesia and 1% intracameral lidocaine (Table 1). The proportion of patients on whom trypan blue, intracameral phenylephrine, or intracameral acetylcholine were used was also similar in the four groups (Table 1). Cefuroxime was predominantly used as an intracameral antibiotic in all four groups (Table 1). The proportion of patients experiencing zonular dehiscence and intraoperative floppy iris syndrome (IFIS) was similar in all four groups (Table 1). The proportion of patients with glaucoma or OHT before surgery ranged between 8 and 9% in three of the groups, but it was lower for the Tecnis Eyhance IOL. These patients were younger, with glaucoma present in only 3.8% of the cases. Consequently, the number of antiglaucoma drugs used in this group was lower than in the other three groups.

**Table 1:** Presurgical characteristics and intraoperative variables of the eyes included in the study.

		B&L® EyeCee One (*n* = 88)	ACU00T0 or SN6CWS Alcon® or AcrySof Toric SN6ATx (*n* = 113)	J&J® Tecnis Eyhance DIB00 (*n* = 106)	PhysIOL® Micropure 123 (*n* = 46)	Total (*n* = 353)
Preoperative characteristics						
Mean age (SD)		74.7 years (6.6 years)	74.5 years (8.6 years)	68.7 years (9.1 years)	69.6 years (8.7 years)	72.5 years (8.7 years)
Male percentage		44 (50%)	35 (31%)	56 (53%)	17 (37%)	152 (43.2%)
Glaucoma/OHT		8 (9.1%)	9 (8%)	4 (3.8%)	4 (8.7%)	25 (7.1%)
Type of glaucoma						
	OHT	1 (1.1%)	3(2.7%)	1 (0.9%)	0	5(1.4%)
	POAG	5 (5.7%)	1 (0.9%)	1 (0.9%)	1 (2.2%)	8 (2.3%)
	PG	0	2 (1.8%)	0	1 (2.2%)	3 (0.8%)
	PEXG	0	2 (1.8%)	1 (0.9%)	0	3 (0.8%)
	Other	2 (2.3%)	1 (0.9%)	1 (0.9%)	2 (4.3%)	6 (1.8%)
	No	80 (90.9%)	104 (92%)	102 (96.2%)	42 (91.3%)	327 (92.9%)
Number of glaucoma drugs (SD)		0.11 (0.03)	0.13 (0.05)	0.05 (0.02)	0.2 (0.1)	0.11 (0.02)
						
IOL power (SD)		22.56 D (2.83 D)	21.45 D (3.98 D)	21.01 D (4.58 D)	20.37 D (7.46 D)	21.46 D (4.58 D)
Axial length (SD)		23.13 mm (0.96 mm)	23.41 mm (1.47 mm)	23.65 mm (1.52 mm)	23.95 mm (2.75 mm)	23.48 mm (1.63 mm)
Anterior chamber Depth (SD)		3.11 mm (0.38 mm)	3.10 mm (0.34 mm)	3.21 mm (0.34 mm)	3.12 mm (0.37 mm)	3.14 mm (0.36 mm)
Presurgical VA (median (IQR))		0.5 (0.33–0.60)	0.33 (0.33–0.50)	0.50 (0.33–0.67)	0.50 (0.33–0.51)	0.46 (0.33–0.6)
VA 1 month after cataract surgery (median (IQR))		0.67 (0.5–1)	0.67 (0.60–0.9)	0.67 (0.6–1)	0.67 (0.5–1)	0.67 (0.5–1)
Nuclear cataract grade [median (IQR)]		3 (3–4)	3 (2.5–3.5)	3 (2.1–3.5)	3 (3–3.5)	3 (2.5–3.5)
Surgical variables:						
Anesthesia	General	1 (1.1%)	2 (1.8%)			3 (0.9%)
	Subtenon	1 (1.1%)	1 (0.9%)	1 (0.9%)		3 (0.9%)
	Topical	86 (98%)	110 (97.3%)	105 (99.1%)	46 (100%)	347 (98.2%)
Trypan blue		12 (13.6%)	18 (16.1%)	15 (14.2%)	2 (4.3%)	47 (13.4%)
Intracameral phenylephrine		39 (44%)	39 (35%)	32 (30%)	19 (41%)	129 (36.7%)
Intracameral acetylcholine		4 (4.5%)	5 (4.5%)	4 (3.8%)	2 (4.3%)	15 (4.3%)
Antibiotic prophylaxis	Cefuroxime	80 (91%)	109 (97%)	99 (93%)	43 (97%)	331 (94%)
	Moxifloxacin	8 (9%)	2 (2%)	6 (6%)	3 (7%)	19 (5.4%)
	Vancomycin	-	1 (1%)	1 (1%)		2 (0.6%)
Zonular disinsertion		2 (2%)	0	1 (1%)	1 (2%)	4 (1.1%)
IFIS		8 (9%)	10 (9%)	5 (4.7%)	3 (6.5%)	26 (7.4%)
Postoperative change in IOP:						
First day	Mean (SD)	7.57 mmHg(8.59 mmHg)	5.13 mmHg (6.28 mmHg)	5.47 mmHg (6.15 mmHg)	5.58 mmHg (6.53 mmHg)	5.83 mmHg (6.87 mmHg)
	Median (IQR)	5 mmHg (2–8.5 mmHg)	4 mmHg (1.75–8 mmHg)	5 mmHg (2–9 mmHg)	5 mmHg (0–10 mmHg)	5 mmHg (2–8.5 mmHg)
	Eyes with IOP rise>5 mmHg	42 (48.3%)	43 (39.8%)	39 (37.5%)	23 (50%)	147 (42.6%)
	Eyes with IOP rise>10 mmHg	18 (20.7%)	15 (13.9%)	15 (14.4%)	11 (23.9%)	59 (17.1%)
						
1 month	Mean (SD)	1.56 mmHg (5.88 mmHg)	−0.53 mmHg (3.89 mmHg)	−1.16 mmHg (3.66 mmHg)	−0.24 mmHg (3.53 mmHg)	−0.18 mmHg (4.48 mmHg)
	Median (IQR)	0 mmHg (−2–3.5 mmHg)	−1 mmHg (−3–2 mmHg)	−1 mmHg (−3–1 mmHg)	0 mmHg (−3–2 mmHg)	0 (−3-2)mmHg
	Eyes with IOP > 5 mmHg	14 (17.1%)	7 (6.7%)	3 (3%)	1 (2.2%)	25 (7.6%)
	Eyes with IOP > 10 mmHg	5 (6.1%)	2 (1.9%)	0	0	7 (2.1%)
						
Cases of NAION		2	0	0	0	2

SD, standard deviation; IQR, interquartile range; OHT, ocular hypertension, VA, visual acuity. AV was measured using a decimal scale.

No significant differences were found in the change in IOP associated with the use of the four IOLs on the first postoperative day (*p* = 0.395), but differences were observed in the change in IOP after 1 month (*p* = 0.016). Contrasts between pairs of IOLs in the IOP change after 1 month showed statistically significant differences between the EyeCee One and the Eyhance IOL (*p* = 0.03) and between the EyeCee One IOL and the Alcon IOL group (ACU00T0 or SN6CWS Alcon® or AcrySof Toric SN6ATx) (*p* = 0.017). However, after applying the Bonferroni correction for multiple comparisons, only the first comparison remained statistically significant (*p* = 0.016).

To achieve greater statistical power, the EyeCee One IOL was compared to the rest of the IOLs. In this analysis, no differences were found on the first postoperative day between the group receiving the EyeCee One implant and those receiving other IOLs. However, differences were observed after 1 month of surgery (*p* = 0.005).

**Figure 1:**
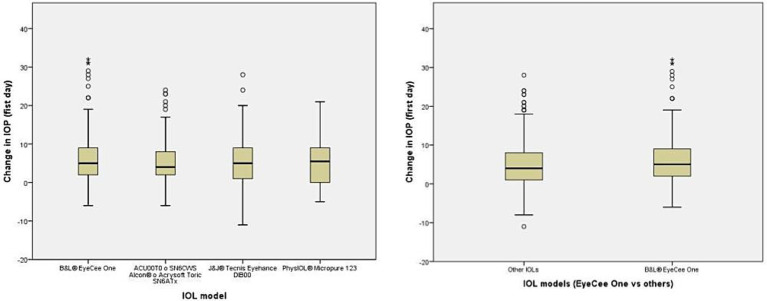
Box plot represents the change in IOP on the first day after the cataract surgery.

**Figure 2:**
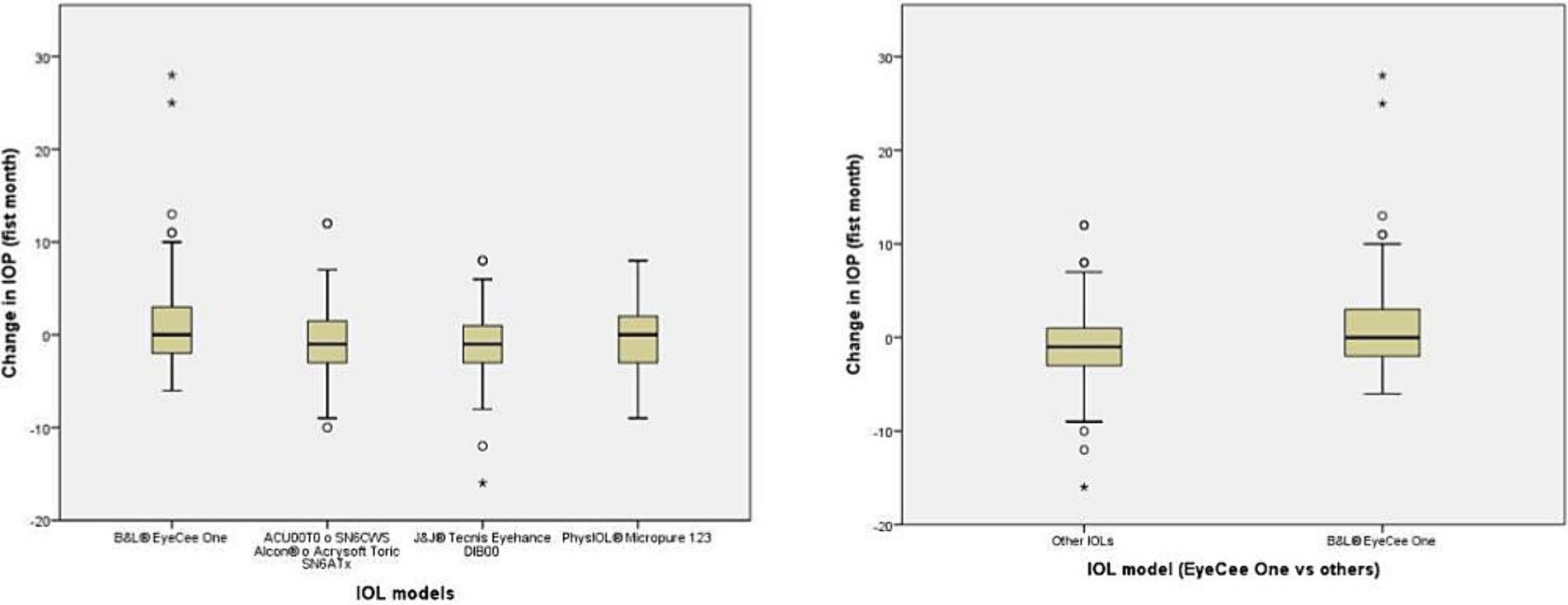
Box plot represents the change in IOP during the first month after cataract surgery.

These groups include patients with higher values of IOP at both postoperative visits (Figures 1, 2). The distribution of IOPS was not symmetrical. The box plot showed the median IOP is higher, the interquartile range is asymmetrical, skewed toward higher values of IOP, and there were several outliers. There was a small group of patients who exhibited a very noticeable hypertensive response, which mostly accounted for the observed differences. (Figures 1, 2). The OR of having at the 1-month visit an IOP elevation higher than 5 mmHg in the EyeCee One patients was 4.44 (1.92–10.22), and the OR of having an IOP elevation higher than 10 mmHg was 7.99 (1.52–41.99) (Table 2).

**Table 2:** Association between IOP rise greater than 5 mmHg and 10 mmHg in the EyeCee One at the first-month visit.

		Change in IOP first day				
		<5 mmHg	>5 mmHg		OR	Range
IOL model	Non-B&L® EyeCee One	237 (95.6%)	11 (4.4%)	248	4.44	(1.92–10.22)
	B&L® EyeCee One	68 (82.9%)	14 (17.1%)	82		
		305 (92.4%)	25 (7.6%)	330		
						
		Change in IOP first month				
		<10 mmHg	>10 mmHg			
IOL model	Non-B&L® EyeCee One	246 (99.2%)	2 (0.8%)	248	7.99	(1.52–41.99)
	B&L® EyeCee One	77 (93.9%)	5 (6.1%)	82		
		323 (97.9%)	7 (2.1%)	330		

The increase in IOP on the first day does not predict the rise in IOP after 1 month; a total of 57 patients had an IOP greater than 10 mmHg the day after surgery; of these, only 2 presented an IOP increase greater than 10 mmHg after 1 month. The OR of showing an increase in IOP greater than 10 mmHg on the first postoperative day and after 1 month was 1.91 (0.36–10.11). Therefore, in our sample, the hypertensive peak on the first postoperative day did not have predictive value for the presence of an elevated IOP after 1 month.

A total of 14 preoperative and intraoperative variables, potentially related to the increase in IOP, were included in a multiple regression model using the backward method, with significance levels of 0.05 for variable inclusion in the model and 0.1 for variable exclusion from the model. The variables included in the model were: age, gender, medical history of glaucoma or intraocular hypertension, nuclear cataract degree, axial length, anterior chamber depth, IOL power, subjective perception of the surgeon of complicated surgery, intraoperative complications, occurrence of IFIS, use of trypan blue, phenylephrine, or acetylcholine, and the use of antibiotic prophylaxis other than the usual (intracameral cefuroxime). The final model included only two variables: the type of IOL (EyeCee One vs. non-EyeCee One), which would be responsible for an increase in IOP between 2 and 3 mmHg; and axial length, which was also associated with higher postoperative IOPs (Table 3).

**Table 3:** Multiple regression model.

Factor	Coefficient (95% CI)	*p*-value
Axial length	0.55 (0.21–0.89)	0.002
IOL model (EyeCee One vs. non-EyeCee One)	2.39 (1.28–3.50)	0.00006

The most significant variable was the type of intraocular lens.

Patients who experienced this IOL-related hypertensive response did not show intraocular inflammation or corneal edema, and gonioscopy revealed no abnormalities. However, two patients suffered severe visual loss among the patients of the EyeCee One group who developed high postoperative IOP. One suffered post-cataract surgery non-arteritic ischemic optic neuropathy (PCSNAION). In the other case, no optic disk swelling was identified, although severe visual loss and severe thinning of the retina’s fiber layer took place over the course of 2 months. No similar cases were observed among the patients who received any of the other IOLs.

## 4 Discussion

In our sample, the implantation of the B&L® EyeCee One IOL was associated with a higher IOP in the postoperative period compared to the other IOLs, both on the first postoperative day and after 1 month. While these differences did not reach statistical significance on the first postoperative day, it is possible that the effect was not observed due to a required incubation period or because the IOL’s effect may have been diluted by various intraoperative factors. These factors include incomplete removal of viscoelastic, surgeries performed by residents, male gender, a history of glaucoma or pseudoexfoliation, axial length greater than 25 mm, poor pupil dilation, and the use of tamsulosin. In the case of the change in IOP at the 1-month visit, the association was statistically significant, although IOP elevation was mild in most cases and the change in IOP was severe in only a few patients. As can be appreciated in the box plots, the majority of patients who received the B&L® EyeCee One IOL exhibited pressure changes similar to those who received other prostheses, with the observed differences being attributed to a small group of patients who displayed an anomalous response. It is challenging to determine whether this variability is due to a true idiosyncratic response or if some unidentified variable is involved.

To determine the type of interaction responsible for this association, preoperative and intraoperative variables were introduced into a multiple regression equation. Two variables were significant: EyeCee One IOL and the axial length. In the logistic regression equation, assuming a linear behavior, each 1 mm increase in axial length would be responsible for a 0.55 mmHg increase in IOP; therefore, it is a small effect from a biological standpoint. This would mean that, after 1 month, an eye with a length of 30 mm would have an IOP approximately 5.5 mmHg higher than an eye with an AL of 20 mm. This effect could be explained by a greater steroid response in myopic patients. Implanting the EyeCee One IOL would result in an increase of almost 2.5 mmHg in IOP, assuming linear behavior, compared to the implantation of other IOLs.

It is interesting to note that neither the perceived subjective complexity of cataract surgery by the surgeon nor the four other variables that could be considered surrogated variables for complexity (nuclear cataract degree, use of trypan blue, phenylephrine, or acetylcholine) were associated with a greater hypertensive response. Furthermore, the hypertensive response does not appear to be related to the use of an intraocular antibiotic other than cefuroxime. Contrary to what was recently published by Jones et al. (1) in our sample, the previous diagnosis of glaucoma or OHT was not associated with a higher risk of developing OHT.

**Figure 3:**
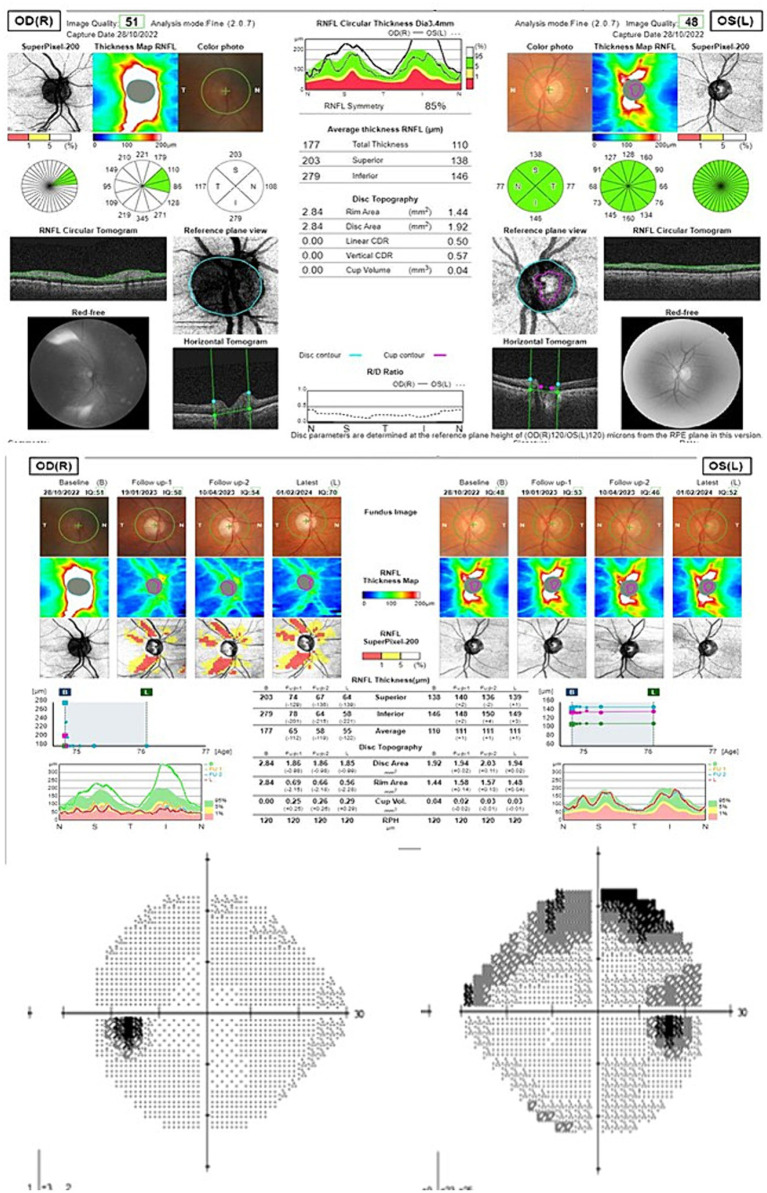
Peripapillary retinal nerve fiber layer OCT and 24–2 Visual field of patient 1 (upper scan at onset, middle evolution over the follow-up time; down: final visual field).

**Figure 4:**
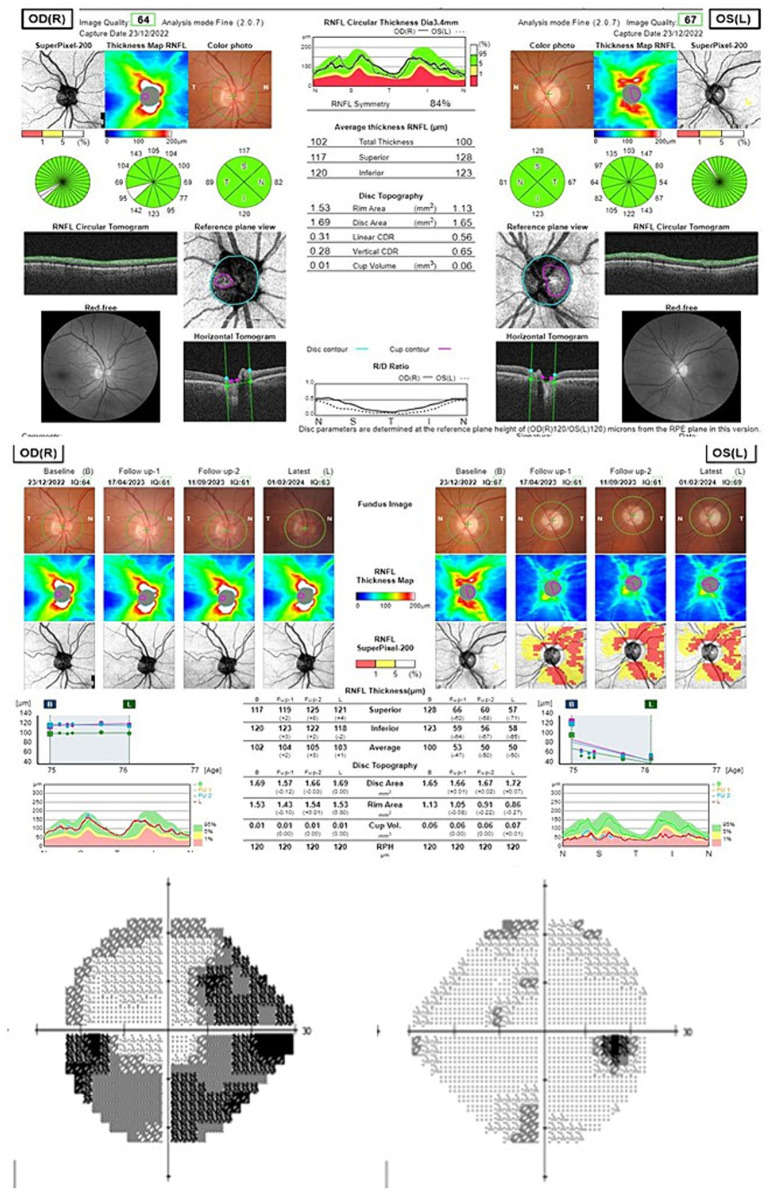
Peripapillary retinal nerve fiber layer OCT and 24–2 Visual field of patient 2 (upper scan at onset, middle evolution over the follow-up time; down: final visual field).

Two patients developed severe visual loss among the group that received the EyeCee One IOL (Table 4). One of them was a clear case of PCSNAION (Figure 3). In the other case (Figure 4), the diagnosis was not so clear (it may have been PCSNAION or just a post-cataract surgery acute glaucoma). It is not possible to test the statistical significance of this finding since, among the 264 patients who received other IOLs, no cases of PCSNAION were detected. However, it is possible to compare these figures with historical data from our center and with data from the literature (5). Prior to these cases, we had only diagnosed four cases of PCSNAION, two of which were reported in a previous article on the morphology of disk at-risk patients (6). Considering that cataract surgery has been performed at our center since 2008 and that we operate 1,500 cataracts each year, the previous incidence of PCSNAION among our patients would be 16.7 PCSNAION per 100,000 cataract surgeries.

**Table 4:** Summary of the two patients that suffered severe visual loss.

	Patient 1 (Figure 3)	Patient 2 (Figure 4)
	Male, 72 years old, right eye	Female, 71 years old, left eye
Past medical history	Psoriasis, HBP	HBP
Glaucoma or family history of glaucoma	No	No
Surgery	Uneventful. Ultrasound time: 2.35 s. Intracameral lidocaine, no intracameral mydriatics, no capsular staining. IOL power 23 (EyeCee One preloaded)	Uneventful. Ultrasound time: 4.01 s. Intracameral lidocaine, no intracameral mydriatics, no capsular staining. IOL power 23.5 (EyeCee One preloaded)
VA	Presurgical: 0.15 Postsrugical: 0.66	Presurgical: 0.65 Postsurgical: 0.95
Cataract grade	NO2	C3NO4
Postsurgical evolution	Visual loss 7 days after surgery. IOP 42 and optic disk edema. Optic disk atrophy 2 months later.	Intense pain did not make possible IOP measurement on the first day visit. Anterior chamber aqueous tap was performed. After aqueous tap IOP was 25. In the first month visit, she referred to visual field loss.
Endothelial count	Presurgical: 1589 Postsurgical: 1552	Presurgical: 2660 Postsurgical: 2043
Postsurgical gonioscopy	Shaffer grade IV, minimum pigmentation of the trabecular meshwork. No synechiae.	Shaffer grade IV, minimum pigmentation of the trabecular meshwork. No synechiae.

HBP-High blood pressure; RE-right eye; LE-left eye.

The incidence of PCSNAION among the 88 patients who were implanted with the EyeCee One would be 2,272 cases in 100,000 (considering both cases as true PCSNAION) or 1,136 in 100,000 (considering only the first case as a true PCSNAION). These incidences are several times higher than the estimated incidence of PCSNAION in our hospital in previous years (16,7 in 100,000), the incidence of PCSNAION in the general population (51.8 in 100,000), and the reported incidence of PCSNAION (7.8 in 100,000) (5) or (10.9 in 100,000) (7). The causal relationship between the IOL and this ischemic event seems plausible, given that the most accepted theories consider that PCSNAION is caused by increased intraoperative or perioperative IOP.

It is notable that the examination of patients who experienced this complication was otherwise normal. Patients who experienced this IOL-related hypertensive response did not show intraocular inflammation or corneal edema, and gonioscopy revealed no abnormalities. Thus, a better understanding of this new form of OHT may be useful in the future in two ways. First, the injection of a high dose of PVP or a related molecule in the anterior chamber may allow for the development of better animal models of glaucoma. Second, it may be useful for the treatment of ocular hypotony.

Animal models of glaucoma are based on genetic selection of individuals with the condition, transgenic animals, obstruction of the trabecular meshwork with particles, or destruction of the trabecular meshwork or episcleral veins (8). Although these models have evolved in recent years, they remain imperfect and unpredictable (8).

Currently, we have an arsenal of drugs and surgical techniques that, with limitations, allow for the treatment of OHT. However, the management of ocular hypotony remains an unresolved issue (9, 10). Serendipity is a very important part of many scientific discoveries. In the history of pharmacology, situations where the identification of a side effect has led to the development of a new drug, potentially useful for treating the opposite condition, are not uncommon. The most well-known case is that of sildenafil in the 1980s (11) but the history of pharmacology is full of similar examples. For instance, the anti-abuse effect of disulfiram was also identified by chance as a side effect when this substance was being researched in the 1940s as a potential anti-scabies drug (12). At present, apart from corticosteroids, ibopamine (13) is the only available drug to treat ocular hypotony, with a very limited effect. A better understanding of the dose-response effect of the substance involved in these increases in IOP may contribute to filling in this gap.

The value of this study is based on the detailed information available for each operated patient and the inclusion of eyes with four different IOL models. The limitations of this study stem from its observational design and the small sample size. In the sample, the distribution of independent variables was similar in all four IOL models (except for age, which was lower in the Tecnis Eyhance DIB00 IOL). However, the choice of IOL could be related to certain clinical or surgical variables that we have not been able to identify and which might be responsible for this atypical evolution. Nevertheless, intraoperative variables are usually associated more with IOP levels on the first postoperative day, and it is more challenging to understand their relationship with IOP levels in the longer term. Studies using larger samples offering greater statistical power are needed to help understand this perplexing response.

In summary, we can conclude that some of the patients who received the B&L® EyeCee One IOL exhibited a more frequent hypertensive response than those who received other IOLs. At this moment, this response could be considered idiosyncratic, as we have not been able to correlate any of the studied variables with this outcome. In our sample, glaucoma patients did not experience greater IOP change.

In the long run, it would be very interesting to identify the mechanism by which the molecule induces OHT and to determine the dose–response curve of this side effect, which in the future could become a therapeutic effect in certain clinical situations.

## Data availability statement

The raw data supporting the conclusions of this article will be made available by the authors, without undue reservation.

## Ethics statement

The study was approved by the Ethics and Clinical Research Committee of the University Hospital of La Princesa (Study Number: 5237) and conducted in accordance with local legislation and institutional requirements. Since this study is retrospective and reports mostly aggregated data, written informed consent was obtained only from the two participants whose data are shown in Table 4 and Figures 3 and 4.

## Author contributions

JGMM: Conceptualization, Data curation, Formal analysis, Funding acquisition, Investigation, Methodology, Project administration, Resources, Software, Supervision, Validation, Visualization, Writing – original draft, Writing – review & editing. YFM: Data curation, Writing – original draft, Writing – review & editing. MCR: Data curation, Writing – original draft, Writing – review & editing. FPG: Data curation, Writing – original draft, Writing – review & editing. VI: Data curation, Writing – original draft, Writing – review & editing. VM: Data curation, Writing – original draft, Writing – review & editing. VPI: Data curation, Writing – original draft, Writing – review & editing. ARP: Data curation, Writing – original draft, Writing – review & editing. RS: Writing – original draft, Writing – review & editing.
